# Untargeted Metabolomics Reveals the Effect of Carbon Dots on Improving the Shelf Life of Postharvest Goji Berries (*Lycium barbarum* L.)

**DOI:** 10.3390/foods14193336

**Published:** 2025-09-26

**Authors:** Yuan-Zhe Wang, Juan Du, Wen-Ping Ma, Run-Hui Ma, Kiran Thakur, Zhi-Jing Ni, Wei Wang, Zhao-Jun Wei

**Affiliations:** 1 School of Biological Science and Engineering, Specialty Food Nutrition and Health Innovation Team of Ningxia Hui Autonomous Region, North Minzu University, Yinchuan 750021, China; 17691150170@163.com (Y.-Z.W.); 20217528@stu.nun.edu.cn (J.D.); zhantingbaiyang@163.com (R.-H.M.); kumarikiran@hfut.edu.cn (K.T.); lovebear@vip.163.com (Z.-J.N.); 2 School of Food and Biological Engineering, Hefei University of Technology, Hefei 230601, China

**Keywords:** fresh wolfberry, carbon dots, photodynamic, postharvest senescence, metabolomics

## Abstract

*Lycium barbarum* L. (goji berry) undergoes rapid quality deterioration after harvest owing to its high water activity and abundant reactive oxygen species (ROS). Carbon-dot-mediated photodynamic treatment (CD-PDT) has recently been shown to extend shelf life by modulating ROS-scavenging and defense enzymes, yet the global metabolic reprogramming that supports this protection remains unresolved. Here, we applied ultra-high-performance liquid chromatography–tandem mass spectrometry (HPLC-MS/MS)-based untargeted metabolomics to decode the metabolic footprint of CD-PDT in freshly harvested goji berries. Our results revealed a total of 17,603 differentially expressed metabolites between the treatment and control groups under both positive- and negative-ion modes. Principal component analysis indicated that CD-mediated PDT significantly altered the metabolic profile of fresh goji berries. The treatment activated the phenylpropanoid biosynthesis pathway, promoting the accumulation of compounds such as kaempferol-3-sophoroside, kaempferol-3-O-β-D-glucoside, and galactoside, thereby enhancing the antioxidant capacity of the fruit. Furthermore, CD-mediated PDT induced the tricarboxylic acid cycle, providing sufficient energy to support the phenylpropanoid biosynthesis pathway. In conclusion, these findings provide the systems-level evidence that CD-PDT orchestrates a coordinated activation of primary and secondary metabolism in postharvest goji berries, establishing a mechanistic framework for preservation of horticultural products.

## 1. Introduction

*Lycium barbarum* L. (goji berry), an orange-red, ellipsoid fruit of the Solanaceae family, is an economically important horticultural crop endemic to north-western Ningxia province, China [1,2]. Renowned for its nutrient-dense matrix, the goji berry possesses significant medicinal value and serves as an important ingredient for functional foods, thereby garnering substantial consumer interest [3]. Goji berries contain a cargo of *L. barbarum* polysaccharides (LBPs) (5–8%) [2], along with abundant carotenoids, polyphenols, and alkaloids that collectively confer a range of physiological activities, including antioxidant, immunomodulatory, hypoglycemic, and anti-aging effects [4,5,6]. Traditionally, goji berries are marketed and consumed as dried fruits; however, the drying process leads to significant degradation of heat-sensitive carotenoids and polyphenols, resulting in a significant loss of nutritional value [7,8]. Consumer demand for minimally processed “superfruits” has therefore shifted attention to fresh-market berries; however, their high respiration rate and thin waxy cuticle render them highly perishable (physiological changes and microbial contamination), which severely limits their practical utility and market expansion [9,10].

Photodynamic therapy (PDT) is an emerging non-thermal sterilization technology that exploits synergism between a photosensitizer (PS), oxygen, and visible light to enhance both the efficacy and the safety of the treatment. It has been widely applied in medical fields such as anti-inflammatory therapy [11] and anticancer treatment [12]. Compared with conventional sanitizing agents, PDT offers negligible microbial resistance, low energy input, and zero chemical residue, positioning it as a sustainable postharvest intervention in the field of food science. While curcumin- and hypericin-mediated photodynamic treatment have demonstrated efficacy in reducing microbial load and delaying senescence in fresh-cut mango, table grapes, and fresh goji berries [13,14,15], their poor aqueous solubility and photobleaching propensity limit translational utility. Shen et al. [14] reported that curcumin-mediated photodynamic processing significantly enhanced the antioxidant capacity of fresh goji berries, reduced oxidative degradation, and improved overall storage quality. He et al. [15] developed a novel multifunctional nanomaterial (CS/CMC@HY) by incorporating the natural photosensitizer hypericin (HY) into a chitosan/carboxymethyl chitosan (CS/CMC) carrier. The development and utilization of novel photosensitizers remain a focal point in photodynamic technology research. Carbon dots (CDs), a class of fluorescent nanomaterials with particle sizes below 10 nm, exhibit excellent visible light absorption, high quantum yield, low toxicity, and ease of surface functionalization, making them candidate next-generation photosensitizers for agri-food applications [16]. Consequently, the rational design of multifunctional, eco-compatible, and cost-effective next-generation photosensitizers has become an urgent imperative for advancing sustainable food-preservation technologies.

Postharvest quality deterioration in fruits is the net manifestation of accelerated primary metabolism, membrane lipid peroxidation, and secondary colonization by opportunistic pathogens. Metabolites serve as direct manifestations of physiological and biochemical changes in fruits [17]. Untargeted metabolomics, especially liquid chromatography–mass spectrometry (LC-MS), enables simultaneous surveillance of >15,000 features, providing a systems-level read-out of biochemical trajectories under different conditions [17,18]. It not only elucidates global metabolic alterations at the molecular level but also facilitates the identification of specific biomarkers, thereby further uncovering the molecular mechanisms underlying fruit quality and health [18]. Integrating metabolomics with chemometrics permits identification of pathway bottlenecks and biomarkers that can be rationally manipulated to extend shelf life.

Our previous research demonstrated that carbon dot-mediated photodynamic therapy (PDT) can delay the senescence process and maintain storage quality of fresh goji berries by modulating antioxidant enzymes and defense-related enzyme activities [19]. Nevertheless, the global metabolic rewiring that reinforces CD-PDT-induced stress tolerance remains unknown. Here, we employed LC-MS-based untargeted metabolomics to construct high-coverage metabolic profiles of CD-PDT-treated versus untreated goji berries throughout different storage periods, and delineate the primary and secondary metabolic pathways that are co-opted to prolong postharvest life. The outcomes will provide mechanistic insight into carbon dot-mediated PDT preservation and guide the rational design of postharvest protocols for functional fresh produce.

## 2. Materials and Methods

### 2.1. Plant Material

Ripe berries of *L. barbarum* L. cultivar “Ningqi No. 1”, the dominant commercial cultivar occupying > 80% of China’s wolfberry acreage, were harvested at full maturity from the National Wolfberry Engineering Research Center, Nanliang Farm, Ningxia Academy of Agriculture and Forestry Sciences, Yinchuan, China (longitude: 106°09′43.41″; latitude: 38°38′42.51″).

### 2.2. Preparation of Carbon Dots

According to a previously reported method [20], citric acid (2.0 g) and ethylenediamine (1.0 mL, ≥99%) were dissolved in 40 mL ultrapure water (18.2 MΩ cm) under magnetic stirring (300 rpm, 5 min). The homogeneous solution was transferred to a 50 mL Teflon-lined stainless-steel autoclave and hydrothermally treated at 150 °C for 2 h. After cooling to room temperature, the crude product was centrifuged (10,000× *g*, 15 min, 25 °C) and the supernatant was filtered through a 0.22 µm PTFE syringe filter (Shanghai, China). The filtrate was dialyzed against ultrapure water [3 kDa MWCO cellulose membrane (Amicon, Shanghai, China), 24 h, RT, three water changes] to remove residual precursors and small-molecule by-products. The purified dispersion was frozen (−80 °C, 12 h) and lyophilized (−50 °C, 0.05 mbar, 48 h) to obtain solid CDs; the resulting powder exhibited high water solubility (>50 mg mL^−1^) and was stored in a desiccator at 4 °C until use.

### 2.3. Photodynamic Treatment of Fresh Lycium barbarum Fruit

According to the orthogonal matrix of Du et al. [19], which established that the efficacy of photodynamic technology in fresh fruit processing primarily depends on the photosensitizer itself rather than the light source, we adopted their optimal CD treatment concentration, optimal light intensity range, and light duration. Briefly, *L. barbarum* berries cv. “Ningqi No. 1” were surface-sterilized in 0.1% (*w*/*v*) NaOCl for 3 min, rinsed three times with sterile distilled water, and air-dried under laminar flow. A uniform layer of carbon-dot (CD) dispersion (0.10 g L^−1^, pH 6.8) was sprayed onto the berry surface (≈0.5 mL g^−1^ fruit) (T group). The fruits were then positioned on a glass platform 20 cm beneath a 450 nm LED array (photon flux density 100 mW cm^−2^) and illuminated for 10 min, turning the fruit every 2.5 min to ensure homogeneous exposure. For the control group (CK), fruits received an equivalent volume of sterile distilled water and were kept in complete darkness. All samples were transferred to ventilated polyethylene crates and stored in the dark at 25 ± 1 °C and 38 ± 2% RH for 15 days. Decay incidence and external quality were recorded every 3 days from day 0. The treatment group and the blank control group were kept consistent in terms of timeline (including storage period and sampling frequency) during the experiment, and finally, the differences in decay severity (decay incidence and external quality) between the two groups were recorded every 3 days from day 0. For each experimental photographic record, images were randomly selected from three independent replicates to ensure representativeness. Berries intended for biochemical analyses were snap-frozen in liquid nitrogen and stored at −80 °C until use.

### 2.4. Determination of Decay Rate

Throughout the 15-day storage period, *L. barbarum* fruits were visually inspected every 3 days for decay symptoms. At each sampling point, three independent replicate experiments were conducted, with 30 fruits evaluated per replicate. A berry was classified as decayed when visible fungal lesions, soft rot, or mycelial growth exceeded 10% of the surface area. During the storage period, the effectiveness of CDs-PDT treatment was determined by comparing the number of decayed fruits between the blank group and the control group.

The decay rate (% *w*/*w*) was calculated as

$$\mathrm{Fruit}\;\mathrm{decay}\;\mathrm{rate}=\frac{\mathrm{number}\;\mathrm{of}\;\mathrm{decayed}\;\mathrm{fruits}}{total\;number\;of\;fruits}\;\times \;100\%$$

### 2.5. Metabolomic Profiling of Fresh Lycium barbarum Fruit

#### 2.5.1. Experimental Workflow

The study of non-targeted metabolomics in this paper includes the following sequential steps: sample collection, quality control sample (QC) preparation, metabolite extraction, UPLC-HRMS analysis, and multivariate statistics principal component analysis (PCA), partial least-squares discriminant analysis (PLS-DA), and bioinformatics (differential-metabolite annotation, KEGG pathway mapping, and hub-metabolite ranking).

#### 2.5.2. Sample Information

Following the treatment protocol of Du et al. [19], berries stored for 9, 12, and 15 days were selected to cover mid- and late-storage senescence windows. Fruits were assigned as follows: control groups: CK9, CK12, CK15; and CDs-PDT groups: T9, T12, T15. Each time-point consisted of six biological replicates (*n* = 6). After calyx removal, whole berries were snap-frozen in liquid nitrogen and stored at −80 °C until analysis.

#### 2.5.3. Metabolite Extraction and QC Preparation

Frozen berries (50 ± 0.5 mg) were homogenized in 2 mL Eppendorf tubes (Thermo Fisher Scientific Inc. Co., Shanghai, China) with 500 µL ice-cold 80% (*v*/*v*) methanol and two 3 mm stainless-steel beads using a cryo-mill (30 Hz, 60 s). The extracts were vortexed, incubated at −20 °C for 30 min to precipitate proteins, and centrifuged (20,000× *g*, 15 min, 4 °C). Supernatants were transferred to LC vials. Since our study needs to exclude external differential factors such as equipment and environment, a pooled QC sample was prepared by combining 50 µL aliquots from every individual extract. In the ultra-performance liquid chromatography–high-resolution mass spectrometry (UPLC-HRMS) analysis sequence, the extracted samples were arranged in a random order for instrumental injection. Quality control (QC) samples were inserted for injection at the beginning, after every 10 analytical injections, and at the end of the sequence, so as to monitor instrument drift, ensure data integrity throughout the entire analysis process, and evaluate the reproducibility of the experiment.

### 2.6. Liquid Chromatography–Mass Spectrometry Analysis

#### 2.6.1. Liquid Phase Conditions

Metabolite separation was performed on an ACQUITY UPLC HSS T3 column (100 mm × 2.1 mm, 1.8 µm particle size, Waters, Milford, MA, USA) thermostatted at 40 °C. A binary solvent system was employed. Eluent A included water (with 5 mmol/L of ammonium acetate and 5 mmol/L of acetic acid) and Eluent B included acetonitrile (LC-MS grade). The gradient profile is detailed in Supplemental Table S1, delivered at a constant flow rate of 0.30 mL min^−1^.

#### 2.6.2. Mass Spectrum Conditions

Metabolites were analyzed on a Q-Exactive HF-X hybrid quadrupole–Orbitrap mass spectrometer (Thermo Fisher Scientific, Bremen, Germany) operated in data-dependent acquisition (DDA) mode with polarity switching (Supplemental Table S2). In one acquisition cycle, the primary acquisition collected the precursor spectrum at 70,000 resolution (70–1050 *m*/*z*), and reached the AGC target 3e6, and the maximum injection time was set to 100 ms. In DDA mode, the first 3 signal ions with an accumulated signal intensity of more than 100,000 were selected for secondary fragmentation scanning, and fragment spectra were collected at a resolution of 17,500, with a maximum injection time of 50 ms and a dynamic exclusion setting of 6 s. A pooled QC sample was injected every 10 analytical runs to monitor signal stability and correct for instrumental drift.

### 2.7. Data Processing and Analysis Methods

The metabolomic analysis in this study was conducted by Zhejiang Lianchuan Biotechnology Co., Ltd. (Hangzhou, China). *L. barbarum* samples, including quality control (QC) samples, were analyzed using HPLC-MS in both positive- and negative-ion modes. The raw mass spectrometry data were converted into mzXML format using MSConvert software (v2023), followed by peak extraction and retention-time correction performed with XCMS software (v4.7). Metabolite identification and quantification of differential metabolites were carried out using metaX software (v2022). Significantly differential metabolites across comparative groups were screened based on fold change (FC) and univariate statistical analysis (*t*-test *p*-value), with selection criteria set as FC ≥ 1.2 or ≤0.8 and *p* < 0.05. Visualization tools such as Hierarchical Cluster Analysis (HCA) diagrams, heatmaps, volcano plots, and Venn diagrams were generated using Origin 2023. Pathway analysis was conducted using the Kyoto Encyclopedia of Genes and Genomes (KEGG) database (https://www.genome.jp/kegg/), with significant metabolic pathways enriched using the KEGG pathway mapper. Metabolite interaction networks were analyzed using MetaboAnalyst 5.0 and visualized with Cytoscape (v3.10.3).

## 3. Results and Discussion

### 3.1. Changes in the Decay Rate and Apparent Changes in Fresh Fruit Wolfberry During Storage

**Figure 1:**
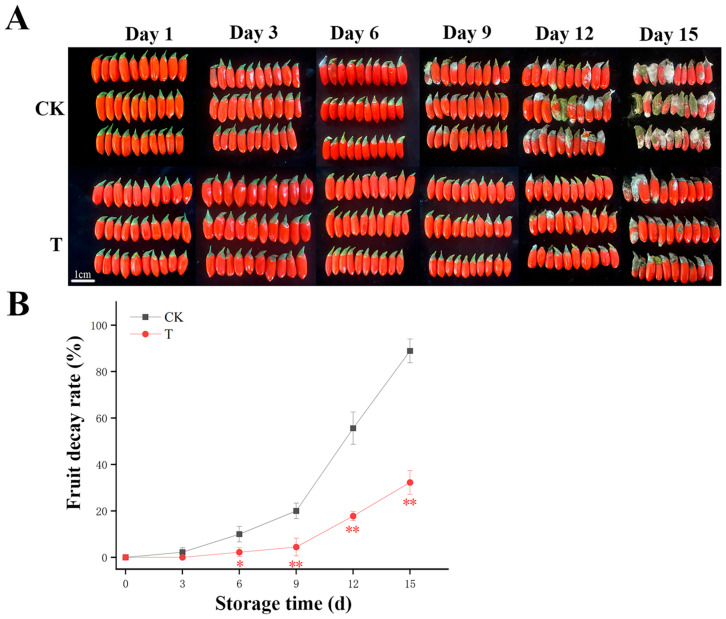
Photographic records (**A**) and quantitative decay kinetics (**B**) of postharvest goji berries (*Lycium barbarum* cv. *Ningqi* No. 1) under CD-mediated photodynamic treatment (T) or control (CK) during 15 days of storage. Data are presented as mean ± standard deviation (mean ± SD) (n = 3). At each sampling time-point, the significance of differences between the control group (CK group) and the treatment group (T group) is marked with * for significant differences (*p* < 0.05) and ** for extremely significant differences (*p* < 0.01), respectively.

During the 15-day storage period, the decay rate of all experimental groups increased, while CDs-PDT-treated *L. barbarum* retained superior visual quality (Figure 1A,B). As shown in Figure 1A, fruits in the control group (CK) initiated decay on day 9, whereas the carbon dot-based photodynamic treatment group (CDs-PDT) exhibited delayed decay onset until day 12. Decay in both groups was triggered by fungal growth. These observations suggest that CDs-PDT treatment may enhance resistance against pathogenic fungi by modulating postharvest physiological metabolism, thereby delaying decay progression. Specifically, the earlier decay in the control group may be associated with oxidative stress imbalance—characterized by reactive oxygen species accumulation leading to membrane lipid peroxidation and increased membrane permeability—as well as insufficient levels of defense-related compounds such as phytoalexins and phenolic compounds [19]. In contrast, CDs-PDT treatment likely maintains intracellular redox homeostasis by enhancing the activity of antioxidant enzymes such as superoxide dismutase and catalase, while simultaneously promoting the synthesis of defense-related metabolites. These physiological alterations are closely linked to metabolomic changes detailed in subsequent sections: the CDs-PDT group showed significantly higher abundances of metabolites associated with plant defense responses (e.g., key intermediates in the phenylpropanoid pathway) and significantly lower abundances of metabolites related to cell wall degradation and oxidative damage. These differential metabolomic profiles support the distinct postharvest physiological states between the two groups, ultimately manifesting as asynchronous decay onset. Quantitatively, decay rates in the CDs-PDT group were 15.6% lower at day 9, 37.8% lower at day 12, and 56.7% lower at day 15, when control decay approached 90%. These outcomes corroborate the photodynamic inactivation mechanism described by Sei et al. [21], whereby 450 nm excited CDs generate cytotoxic Reactive Oxygen Species (ROS) that suppress spoilage-associated pathogens, while concomitant oxidative priming up-regulates phenylpropanoid metabolism, enhances endogenous antioxidants, and thereby delays microbial-mediated fruit deterioration. Furthermore, prior research by Du et al. on the toxicity assessment of carbon quantum dots demonstrated that their application in fresh goji berry processing does not induce any adverse effects [19].

### 3.2. QC-Based Total-Ion-Current Chromatogram Reproducibility

**Figure 2:**
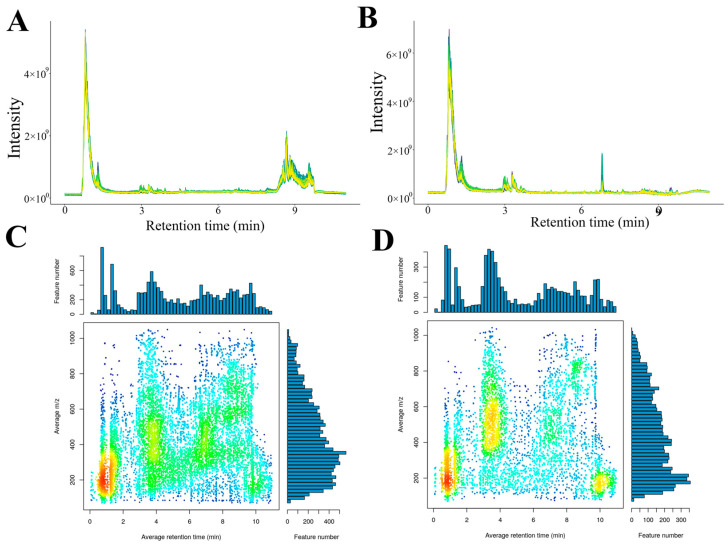
QC consistency and feature distribution. Overlapping TIC profiles of QC samples in positive (**A**) and negative (**B**) modes. Distribution of *m*/*z*-rt under positive mode (**C**) and negative mode (**D**).

Figure 2 presents the superimposed total-ion-current (TIC) chromatograms obtained from the pooled QC samples. In both positive (Figure 2A) and negative (Figure 2B) electrospray-ionization modes, the TIC traces exhibited nearly identical retention-time distributions and peak-intensity patterns, with coefficients of variation for the 10 most abundant features < 4%. The high degree of spectral overlap confirmed the excellent instrumental stability and analytical reproducibility, thereby validating the dataset for subsequent chemometric analysis.

This study employed XCMS software to perform peak alignment processing on mass spectrometry data, thereby enabling more accurate quantification of metabolites. As shown in Figure 2C,D, >80% of these ions accumulated between *m*/*z* 200 and 600 and eluted within 0–10 min, indicating a predominance of low-molecular-weight, polar metabolites amenable to early-gradient RP-UHPLC separation (Figure 2).

### 3.3. Metabolite Clustering and Quality-Assurance Validation of QC Samples

**Figure 3:**
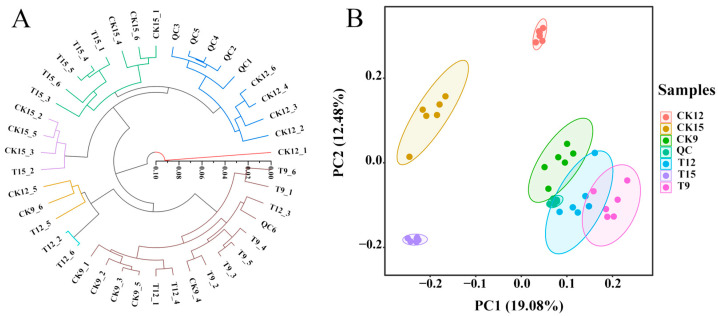
Unsupervised clustering and dimension-reduction validation of metabolomic data in positive- and negative-ion modes. HCA heatmap (**A**) and PCA score plot (**B**).

To validate analytical reproducibility and inter-group separation, unsupervised HCA and PCA were performed on the total-ion-current matrices obtained from QC, CK (CK9, CK12, CK15), and CDs-PDT (T9, T12, T15) samples (Figure 3). HCA revealed three discrete clusters, namely CK, T, and QC, each exhibiting tight intra-group linkage distances and well-defined separation between cohorts (Figure 3A). PCA showed a clear segregation along PC1 (19.08% of total variance) and PC2 (12.48%), with QC samples forming a compact centroid and CK/T samples occupying distinct quadrants (Figure 3B). The absence of within-group dispersion and the pronounced between-group separation confirm high repeatability and demonstrate that the metabolomic dataset is sufficiently robust for downstream differential-metabolite and pathway analyses.

### 3.4. Metabolite Identification and Annotation

#### 3.4.1. Identification of Metabolites

Untargeted metabolomic profiling was performed in both positive and negative electrospray-ionization modes. Features were first de-replicated against an in-house spectral library containing MS^1^ and MS^2^ records of authentic standards acquired under identical LC-HRMS conditions. Remaining ions were annotated against public databases including KEGG, HMDB, and METLIN using the following stringent matching criteria: exact mass tolerance ≤ 25 ppm, retention-time deviation ≤ 30 s, and MS^2^ spectral similarity ≥ 0.3 cosine score. Confidence levels were assigned according to the Metabolomics Standards Initiative (MSI) guidelines. In POS mode, a total of 12,231 metabolites were identified, among which 5656 (KEGG) and 6355 (HMDB) substances were identified by primary spectrogram, and 220 substances were identified by secondary spectrogram (Supplemental Table S3). Under the NEG model, 5372 metabolites were identified, among which 2449 (KEGG) and 2690 (HMDB) species were identified by primary spectrogram, and 230 species were identified by secondary spectrogram (Supplemental Table S3).

#### 3.4.2. Metabolite Identification and KEGG Annotation

**Figure 4:**
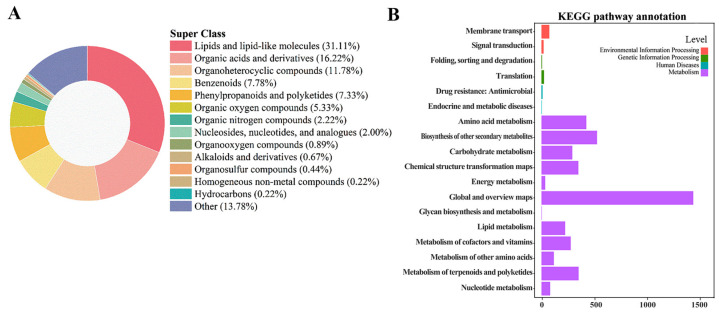
Metabolite classification and pathway mapping of the *Lycium barbarum* fruit metabolome following CD-PDT. Hierarchical Cluster Analysis (HCA): (**A**) super class distribution of high-confidence features across three biological replicates per time-point and two treatments (Ctrl vs. CD-PDT). KEGG pathway-level annotation: (**B**) the top-enriched modules are phenylpropanoid biosynthesis, lipid metabolism, and carbohydrate metabolism, supporting the observed antioxidant enhancement and energy supply under CD-PDT.

MS/MS-confirmed metabolites (450 entities) were first classified into six dominant chemical families: lipids and lipid-like molecules dominated at 31.1%, followed by organic acids and their derivatives (16.2%), heterocyclic compounds (11.8%), phenyl-containing molecules (7.9%), phenylpropanoids and polyketides (7.3%), and organooxygen compounds (5.3%) (Figure 4A). Subsequent KEGG annotation (Figure 4B) revealed that the majority (≈68%) of these metabolites participate in core metabolic processes, including amino acid metabolism, biosynthesis of other secondary metabolites, carbohydrate metabolism, energy metabolism, glycan biosynthesis and metabolism, lipid metabolism, metabolism of cofactors and vitamins, metabolism of other amino acids, terpenoid and polyketide metabolism, and nucleotide metabolism.

### 3.5. PLS-DA Model and Score Analysis Between Groups

**Figure 5:**
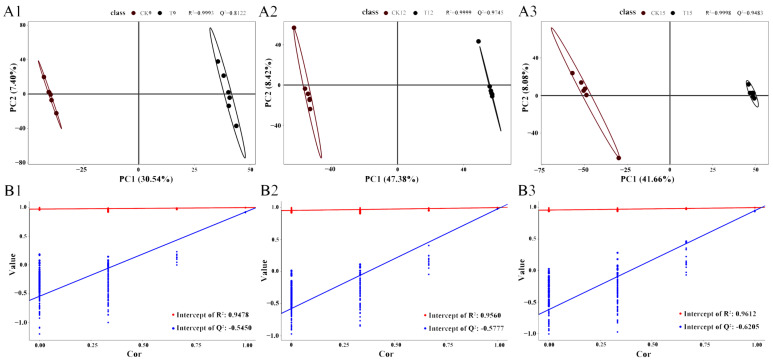
PLS-DA model diagnostics across storage time-points (CK vs. T). PLS-DA (**A1**–**A3**) score and adjoint displacement test of comparison groups (**B1**–**B3**).

A supervised partial least-squares discriminant analysis (PLS-DA) was constructed to correlate sample class (CK vs. T) with metabolite expression patterns. Model performance was evaluated by the goodness-of-fit parameter R^2^Y (explained variance) and the predictive ability parameter Q^2^ (cross-validated R^2^). A robust model is indicated when R^2^Y approaches 1 and Q^2^ > 0.5. All pairwise comparisons (CK9 vs. T9, CK12 vs. T12, CK15 vs. T15) satisfied these thresholds (R^2^Y ≈ 0.99, Q^2^ > 0.7; Figure 5(A1,B1)). In the corresponding score plots, CK samples aligned on the left of component 1, whereas T samples clustered on the right, demonstrating clear metabolomic separation. Permutation tests (200 random label rearrangements) confirmed model validity: regression intercepts of Q^2^ were consistently < 0, and R^2^ intercepts were markedly lower than their respective model R^2^ values (Figure 5(A2,A3,B2,B3)), indicating an absence of overfitting. Collectively, the PLS-DA model was statistically sound and suitable for downstream differential-metabolite and pathway analyses.

### 3.6. PCA Between Groups and Analysis of Major Differential Metabolites

**Figure 6:**
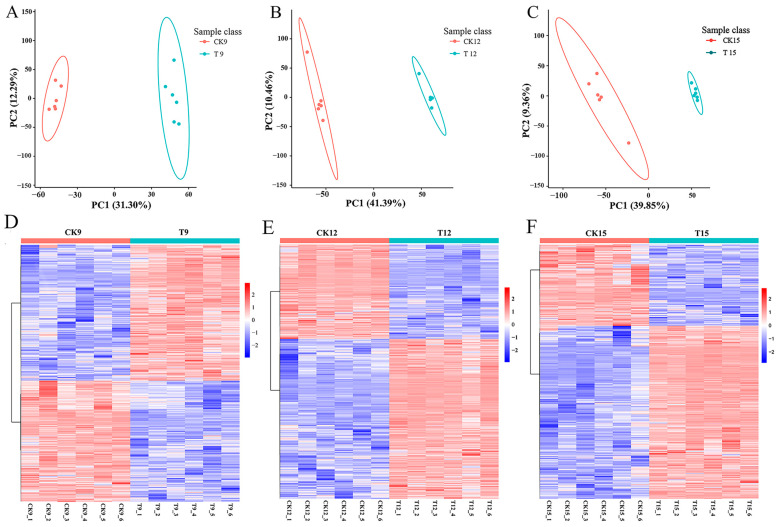
PCA diagram of comparison groups and differential-metabolite heatmaps for each comparison group: T9 vs. CK9 (**A**,**D**), T12 vs. CK12 (**B**,**E**), T15 vs. CK15(**C**,**F**).

PCA of the untargeted LC–MS datasets revealed a clear progressive shift in the metabolic signatures of CDs-PDT-treated wolfberries relative to untreated controls (Figure 6A). On day 9 (T9 vs. CK9), PC1 and PC2 jointly captured 43.59% of the total variance, with PC2 contributing the majority (31.30%), indicating that early metabolic divergence was driven by secondary components. By day 12 (T12 vs. CK12), PC1 became dominant, accounting for 43.39% of the variance while PC2 dropped to 10.46%, reflecting the emergence of a strong, treatment-correlated metabolic trajectory. This trend intensified on day 15 (T15 vs. CK15), where PC1 explained 39.85% and PC2 9.36%, and the score plots exhibited maximal separation between groups, confirming that the metabolic distance between treated and control fruits increased continuously throughout storage.

To identify the metabolites underlying this divergence, we applied a combined filter of FC ≥ 1.2 (or ≤0.83) and *t*-test *p* < 0.05. The heatmap (Figure 6D–F) showed that the T group consistently exhibited more up-regulated than down-regulated ions across both ionization modes, with the greatest imbalance observed on day 15. The most responsive metabolite classes included phenylpropanoids, particularly flavonoids and phenolic acids such as glycerophospholipids, di- and triglycerides, sugars such as fructose-6-phosphate and sucrose, free amino acids (proline, valine, γ-aminobutyric acid), and intermediates of the TCA cycle (citrate, α-ketoglutarate).

Combined with the results of Ge et al., it can be seen that the phenylpropanoid metabolic pathway is one of the most important secondary metabolic pathways, which can improve the antioxidant capacity of plants, and this conclusion provides some theoretical support for this paper [22].

### 3.7. Total Metabolite Analysis of CDs-PDT-Treated Wolfberry Fruits

**Figure 7:**
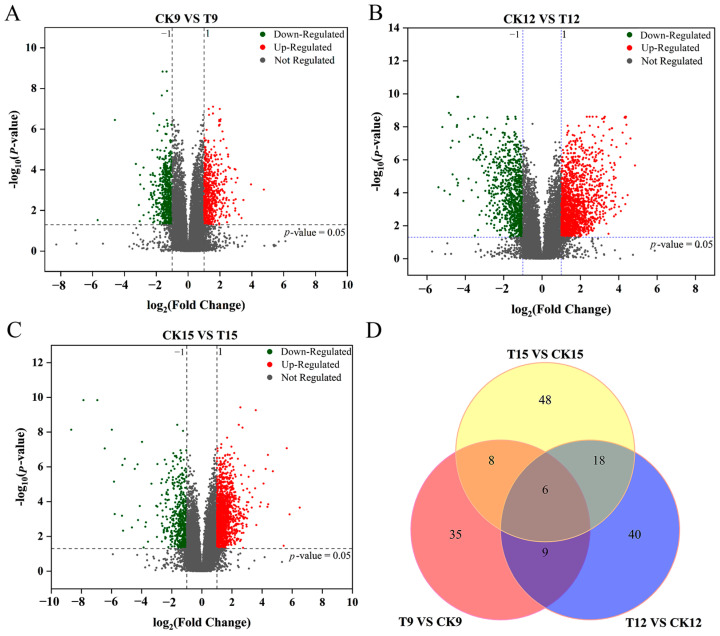
Volcano and Venn analyses of CDs-PDT-induced metabolomic shifts in postharvest wolfberry fruits. Volcano plots (**A**–**C**) for the differential ion features detected in positive- and negative-ion modes for each pairwise comparison. (**A**) T9 vs. CK9 (after 9 d storage), (**B**) T12 vs. CK12 (after 12 d storage), (**C**) T15 vs. CK15 (after 15 d storage). Venn diagram of significantly altered ions across the three storage intervals (**D**).

Untargeted LC–MS profiling captured totals of 3264, 3837, and 3777 high-quality ion features (positive + negative modes) for T9 vs. CK9, T12 vs. CK12, and T15 vs. CK15, respectively (Figure 7). A progressive increase in both the absolute number and the proportion of up-regulated ions was observed over the 15-day storage period: 1726 (52.9%) up- versus 1538 (47.1%) down-regulated ions on day 9; 2409 (62.8%) up- versus 1428 (37.2%) down-regulated ions on day 12; and 2566 (68.0%) up- versus 1211 (32.0%) down-regulated ions on day 15. The trend indicated a cumulative and intensifying metabolic activation elicited by CD-mediated photodynamic treatment.

Venn analysis further resolved these differential ions into 132 treatment-specific metabolites and only six ions common to all three pairwise comparisons (Figure 7D). The exceptionally low overlap (≤0.2%) emphasized the pronounced time-dependency of the metabolic response and suggested that discrete molecular programs were engaged at each storage interval. Collectively, these data provide a comprehensive temporal atlas of CDs-PDT-induced metabolomic reprogramming, offering a robust foundation for subsequent pathway-centric investigations and biomarker discovery.

### 3.8. Temporal Profiling of Key Differential Metabolites Across T9 vs. CK9, T12 vs. CK12, and T15 vs. CK15

**Figure 8:**
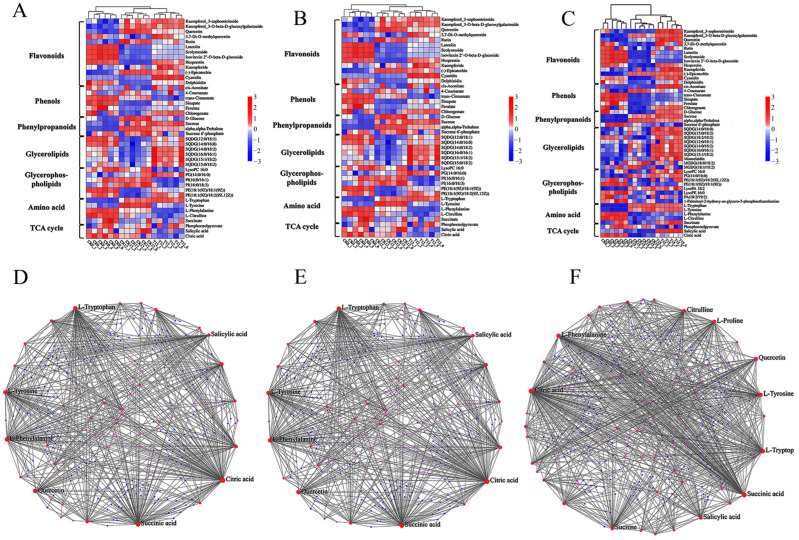
Temporal metabolite fingerprints and interaction networks of fresh goji berries under CDs-PDT. Heatmaps (**A**–**C**) display the scaled abundance (Z-score) of the 50 most variable metabolite ions detected in positive-ion mode (**A**) and negative-ion mode (**B**,**C**) across CK0 (pre-storage baseline), CK9, and T9. Positive-ion flavonoids and phenylpropanoids (**A**), negative-ion glycerolipids/glycerophospholipids (**B**), negative-ion TCA-cycle intermediates (**C**). Interaction networks (**D**–**F**) among the differential metabolites. Node size is proportional to the number of connections (degree centrality); edge thickness reflects correlation strength. Core hub metabolites (salicylic acid, quercetin, sucrose, citric acid, succinic acid, L-phenylalanine, L-citrulline, L-tyrosine, proline, L-tryptophan) are marked in bold.

Across the three storage intervals examined (T9 vs. CK9, T12 vs. CK12, and T15 vs. CK15), CD-mediated photodynamic treatment induced a coherent and time-dependent reprogramming of the wolfberry metabolome that consistently favored the retention of phenylpropanoid and TCA-cycle metabolites relative to the untreated controls. On day 9, the treatment elevated kaempferol-3-O-sophorotriglucoside, kaempferol-3-O-β-D-glucogalactoside, quercetin, 3,7-di-O-methylquercetin, and rutin by 0.8- to 1.4-fold (log_2_ scale) while attenuating the senescence-associated decline in luteolin, scoparone, isovitexin-2-O-β-D-glucoside, and naringenin; concurrent preservation of citrate and α-ketoglutarate indicated an early deceleration of respiratory losses. By day 12, the same flavonoid signature remained 1.5- to 2.1-fold higher in the treated fruit, and phenolic acids including chlorogenic, ferulic, and salicylic acids were maintained at concentrations statistically indistinguishable from the pre-storage baseline (Figure 8). The most pronounced divergence occurred on day 15 (Figure 8C): treated berries accumulated kaempferol glycosides, quercetin and its methylated congeners, naringenin, kaempferol, epicatechin, and delphinidin at 2.3- to 3.0-fold above the controls, and while both groups experienced decreased 4-coumarate, trans-cinnamate and succinate, the CDs-PDT cohort simultaneously restored ferulic, salicylic, and chlorogenic acids to near-baseline levels. Metabolite–metabolite interaction networks identified salicylic acid, quercetin, sucrose, citrate, succinate, L-phenylalanine, L-citrulline, L-tyrosine, proline, and L-tryptophan as the most highly connected nodes, with elevated phenylalanine serving as the principal entry point into the phenylpropanoid pathway (Figure 8D–F). Collectively, these findings demonstrate that CDs-PDT sustains biosynthetic flux toward flavonoids and hydroxycinnamic acids, thereby reinforcing antioxidant capacity and delaying postharvest senescence while preserving the characteristic nutritional and medicinal quality of fresh goji berry fruit [23].

### 3.9. KEGG-Based Metabolic Pathway Enrichment of T9 vs. CK9, T12 vs. CK12, and T15 vs. CK15

**Figure 9:**
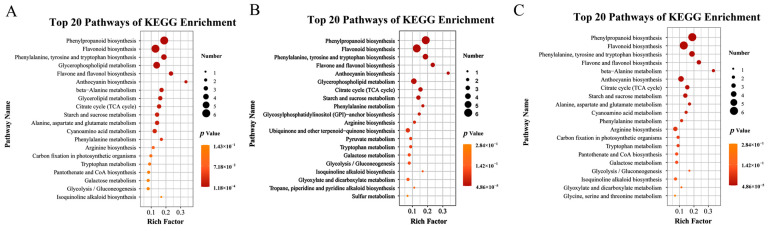
KEGG pathway enrichment of differential metabolites generated by CDs-PDT. Bubble plots display the top 20 most significantly enriched KEGG pathways (ranked by *p*-value) for each pairwise comparison. T9 vs. CK9 (9 d storage) (**A**), T12 vs. CK12 (12 d storage) (**B**), T15 vs. CK15 (15 d storage) (**C**).

KEGG pathway enrichment analyses (Figure 9) showed that the differential metabolites were significantly over-represented in 42 pathways for T9 vs. CK9, 35 for T12 vs. CK12, and 42 for T15 vs. CK15, with phenylpropanoid biosynthesis, flavonoid biosynthesis, phenylalanine/tyrosine/tryptophan biosynthesis, flavone and flavanol biosynthesis and, at specific time-points, anthocyanin biosynthesis (T12) or β-alanine metabolism (T15) emerging as the most enriched routes (*p* < 0.01; Figure 9A–C). These pathways constitute a tightly linked network in which phenylalanine acts as the primary precursor for the phenylpropanoid pathway, supplying carbon skeletons for the downstream production of flavonoids, phenolic acids, and anthocyanins [24]. During postharvest storage, endogenous respiration and stress signaling continuously remodel primary-metabolite pools, thereby activating the above secondary-metabolite routes and triggering a systemic defense response in the fruit [25]. The phenylpropanoid pathway is a well-established hub of plant and fruit disease resistance [26]; its flavonoid, anthocyanin, isoflavone, flavone, and flavanol branches collectively generate coumarins, flavonoids, and phenolic acids that regulate plant development and defense and confer anticancer and anti-inflammatory benefits to humans [27]. Flavonoid biosynthesis, in particular, scavenges excess ROS elicited by biotic or abiotic stress and inhibits microbial spore germination [28], germ-tube elongation, and mycelial growth, thereby providing broad antioxidant and antimicrobial activities [29]. Collectively, CD-mediated photodynamic treatment elevates endogenous phenylalanine, channels sustained carbon flux through the phenylpropanoid pathway, enhances the accumulation of flavonoids and phenolic acids, increases overall antioxidant capacity, and effectively delays postharvest senescence while preserving the freshness and nutritional quality of goji berry fruits [30].

### 3.10. Analysis of KEGG Metabolic Network

**Figure 10:**
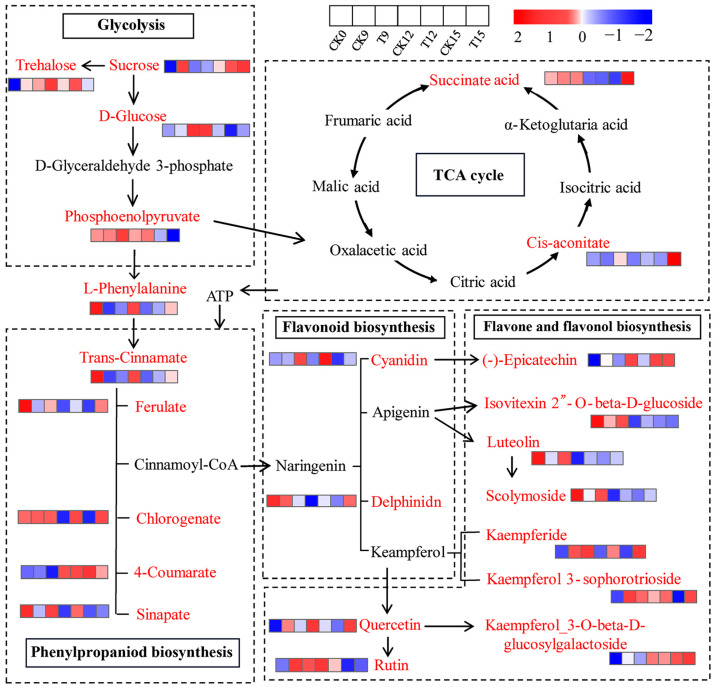
Integrated KEGG metabolic network of CDs-PDT-responsive metabolites in wolfberry fruits. The color red denotes essential metabolites, while black indicates differential metabolites. The abundance levels of differential metabolites are visualized using a heatmap, with the color gradient from blue to red representing increasing metabolite concentrations. Edge style: Solid arrows indicate enzymatic conversions supported by KEGG orthologues; arrow thickness is proportional to reaction confidence.

To elucidate how CDs-PDT orchestrated postharvest metabolism of wolfberry fruits, we merged the significantly altered metabolites with the KEGG database to reconstruct a dynamic metabolic network (Figure 10). Sucrose, the primary soluble carbohydrate imported into the berry, is sequentially hydrolyzed to trehalose and D-glucose and ultimately channeled through glycolysis to yield phosphoenolpyruvate (PEP). PEP then bifurcates: one branch fuels the TCA cycle via pyruvate and acetyl-CoA, while the second branch donates carbon to the phenylpropanoid pathway, leading to the de novo synthesis of trans-cinnamic acid, 4-coumaric acid, ferulic acid, chlorogenic acid, and erucic acid. Activated by 4-coumaroyl-CoA ligase, these hydroxycinnamic acids condense with malonyl-CoA to form naringenin chalcone, which is isomerized to naringenin (the gateway flavanone) and then diversified into anthocyanins (delphinidin, cyanidin), flavones (apigenin, luteolin) and flavonols (kaempferol, quercetin) through flavone synthase and flavanol synthase reactions.

A continuous supply of ATP and reducing equivalents is essential for these energetically costly biosynthetic steps. The TCA cycle serves as the central mitochondrial hub that converts PEP-derived pyruvate into succinate, citrate, cis-aconitate, and α-ketoglutarate while generating NADH and FADH_2_ to fuel oxidative phosphorylation. CDs-PDT significantly elevated the intracellular concentrations of succinate, citrate, cis-aconitate, and PEP on day 9, followed by a transient decline on day 12. It is possible that under conditions where oxidative phosphorylation is impaired due to spoilage, pyruvate is channeled into the alcohol fermentation pathway, with a secondary rise on day 15. This biphasic pattern mirrors the fluctuating energy demand imposed by the fruit’s defense response: when visible mold incidence peaked on day 12, the TCA-cycle intermediates were rapidly consumed to fuel accelerated phenylpropanoid flux and ROS detoxification. Once the stress was countered, metabolite pools re-accumulated by day 15, restoring cellular redox homeostasis.

Accordingly, CDs-PDT sustains the TCA cycle, ensuring that phenylalanine-driven phenylpropanoid biosynthesis receives sufficient ATP and NAD(P)H for flavonoid and phenolic acid accumulation. This metabolic reprogramming enhances the antioxidant capacity of wolfberry fruits, delays senescence, and preserves freshness and storage quality. Yan et al. [31] demonstrated that methyl jasmonate similarly boosts TCA-cycle output to energize phenylpropanoid defenses against Botrytis cinerea in blueberry, while Wang et al. [32] showed that exogenous phenylalanine delays pear aging by reinforcing the TCA cycle [33]. Collectively, our findings indicate that CDs-PDT acts as a light-activated metabolic modulator that couples carbon and energy metabolism to secondary-metabolite defense, thereby extending the postharvest life of the goji berry.

## 4. Conclusions

This study employs widely targeted metabolomics to systematically investigate the metabolic profile of postharvest fresh goji berry fruits and explores the impact of carbon dot-mediated photodynamic therapy (PDT) on their metabolic levels. Our results indicate that carbon dot-mediated PDT enhanced phenylpropanoid metabolism in wolfberry fruits by promoting the accumulation of various phenylpropanoid metabolites, including quercetin, 3,7-di-O-methylquercetin, naringenin, kaempferol, epicatechin, delphinidin, ferulic acid, and chlorogenic acid. Additionally, carbon dot-mediated PDT stimulated the tricarboxylic acid cycle to provide energy for the phenylpropanoid biosynthesis pathway, thereby maintaining high levels of flavonoids and phenolic compounds in the fruits. This process could enhance the antioxidant capacity of wolfberry fruits, reduce cellular oxidative damage, and delay fruit senescence. Our current study used a single cultivar (Ningqi No. 1) and a fixed CD-PDT dose; therefore, genotype- and dose-dependent metabolic responses remain untested. Integrating the current metabolome with forthcoming transcriptome and proteome datasets will enable construction of a multi-layered regulatory network, clarifying how nano-enabled photodynamic signaling is transduced into quality-related biochemical phenotypes. The findings establish a mechanistic platform for precision, non-thermal preservation of goji berries.

## Data Availability

The original contributions presented in the study are included in the article/Supplementary Material, further inquiries can be directed to the corresponding author.

## Supplementary Materials

The following supporting information can be downloaded at https://www.mdpi.com/article/10.3390/foods14193336/s1: Table S1. Liquid phase gradient setting parameters; Table S2. Mass spectrum parameters; Table S3. Differential metabolites and identification results; Table S4. CK9vsT9_sample_data; Table S5. CK12vsT12_sample_data; Table S6. CK15vsT15_sample_data.

## References

[1] Ma R.H., Zhang X.X., Thakur K., Zhang J.G., Wei Z.J. (2022). Research progress of *Lycium barbarum* L. as functional food: Phytochemical composition and health benefits. Curr. Opin. Food Sci..

[2] Wang W., Ni Z.J., Song C.B., Ma W.P., Cao S.Q., Wei Z.J. (2023). Hydrogen sulfide treatment improves quality attributes via regulating the antioxidant system in goji berry (*Lycium barbarum* L.). Food Chem..

[3] Amagase H., Farnsworth N.R. (2011). A review of botanical characteristics, phytochemistry, clinical relevance in efficacy and safety of *Lycium barbarum* fruit (Goji). Food Res. Int..

[4] Potterat O. (2010). Goji *(Lycium barbarum* and *L. chinense*): Phytochemistry, pharmacology and safety in the perspective of traditional uses and recent popularity. Planta Med..

[5] Ni Z.J., Liu C.B., Xue Y., Huang H., Ma Y.L., Thakur K., Shang Y.F., Khan M.R., Wei Z.J. (2025). Enhanced protection and bioavailability of *Lycium barbarum* leaf extract through encapsulation in whey protein isolate and bovine serum albumin nanoparticles. Food Chem..

[6] Gong G., Dang T., Deng Y., Han J., Zou Z., Jing S., Zhang Y., Liu Q., Huang L., Wang Z. (2018). Physicochemical properties and biological activities of polysaccharides from *Lycium barbarum* prepared by fractional precipitation. Int. J. Biol. Macromol..

[7] Fratianni A., Niro S., Alam M.D.R., Cinquanta L., Di Matteo M., Adiletta G., Panfili G. (2018). Effect of a physical pre-treatment and drying on carotenoids of goji berries (*Lycium barbarum* L.). LWT.

[8] Ma Z.F., Zhang H., Teh S.S., Wang C.W., Zhang Y., Hayford F., Wang L., Ma T., Dong Z., Zhang Y. (2019). Goji Berries as a Potential Natural Antioxidant Medicine: An Insight into Their Molecular Mechanisms of Action. Oxidative Med. Cell. Longev..

[9] Hu X.X., Sun H., Yang X.D., Cui D.J., Wang Y.Q., Zhuang J., Wang X.X., Ma R.N., Jiao Z. (2021). Potential use of atmospheric cold plasma for postharvest preservation of blueberries. Postharvest Biol. Technol..

[10] Cong K.P., Li T.T., Wu C.E., Zeng K.F., Zhang J.H., Fan G.J., Pan Y., Wang J.H., Suo A.D. (2022). Effects of plasma-activated water on overall quality of fresh goji berries during storage. Sci. Hortic..

[11] Kabakov A.V., Kazakov O.V., Poveshchenko A.F., Cherkas V.N., Kononchuk V.V. (2024). Influence of Photodynamic Therapy with Subsequent Surgical Treatment of Experimental Breast Cancer on Quantitative Changes in miRNAs in a Mesenteric Lymph Node. Bull. Exp. Biol. Med..

[12] Soares da Silva N., Ferreira-Strixino J., Pacheco-Soares C. (2024). Photodynamic therapy: Challenges and innovations for treating cancer. Front. Oncol..

[13] Pramana A., Firmanda A., Arnata I.W., Sartika D., Sari E.O. (2024). Reduction of biofilm and pathogenic microorganisms using curcumin-mediated photodynamic inactivation to prolong food shelf-life. Int. J. Food Microbiol..

[14] Shen Y.F., Ma W.P., Ma R.H., Thakur K., Ni Z.J., Wang W., Wei Z.J. (2025). Curcumin-Mediated Photodynamic Treatment Enhances Storage Quality of Fresh Wolfberries via Antioxidant System Modulation. Foods.

[15] He J., Zhang S., Hu Z., Chen H., Wang Y., Feng K., Yao J., Yuan Y., Yue T., Sheng Q. (2025). Antibacterial photodynamic inactivation of pH-responsive microspheres based on chitosan/carboxymethyl chitosan with hypericin and its application in food preservation. Food Biosci..

[16] Zhang Y., Li P., Su R., Wen F., Jia Z., Lv Y., Cai J., Su W. (2022). Curcumin-loaded multifunctional chitosan gold nanoparticles: An enhanced PDT/PTT dual-modal phototherapeutic and pH-responsive antimicrobial agent. Photodiagnosis Photodyn. Ther..

[17] Tian X., Zhu L., Yang N., Song J., Zhao H., Zhang J., Ma F., Li M. (2021). Proteomics and metabolomics reveal the regulatory pathways of ripening and quality in post-harvest kiwifruits. J. Agric. Food Chem..

[18] Chen Y.S., Li J., Menon R., Jayaraman A., Lee K., Huang Y., Dashwood W.M., Zhang K., Sun D., Dashwood R.H. (2021). Dietary spinach reshapes the gut microbiome in an Apc-mutant genetic background: Mechanistic insights from integrated multi-omics. Gut Microbes.

[19] Du J., Ni Z.J., Wang W., Thakur K., Ma R.H., Ma W.P., Wei Z.J. (2024). Carbon Dot-Mediated Photodynamic Treatment Improves the Quality Attributes of Post-Harvest Goji Berries (*Lycium barbarum* L.) via Regulating the Antioxidant System. Foods.

[20] Liu S., Cui J., Huang J., Tian B., Jia F., Wang Z. (2019). Facile one-pot synthesis of highly fluorescent nitrogen-doped carbon dots by mild hydrothermal method and their applications in detection of Cr(VI) ions. Spectrochim. Acta Part A Mol. Biomol. Spectrosc..

[21] Seididamyeh M., Netzel M.E., Mereddy R., Sultanbawa Y. (2024). Curcumin-mediated photodynamic treatment to extend the postharvest shelf-life of strawberries. J. Food Sci..

[22] Ge W., Xin J., Tian R. (2023). Phenylpropanoid pathway in plants and its role in response to heavy metal stress: A review. Chin. J. Biotechnol..

[23] Gao Y., Wei Y., Wang Y., Gao F., Chen Z. (2017). *Lycium barbarum*: A Traditional Chinese Herb and A Promising Anti-Aging Agent. Aging Dis..

[24] Sonnante G., D’Amore R., Blanco E., Pierri C.L., De Palma M., Luo J., Tucci M., Martin C. (2010). Novel hydroxycinnamoyl-coenzyme A quinate transferase genes from artichoke are involved in the synthesis of chlorogenic acid. Plant Physiol..

[25] Habibi F., Boakye D.A., Chang Y., Casorzo G., Hallman L.M., Madison M., Clavijo-Herrera J., Sarkhosh A., Liu T. (2024). Molecular mechanisms underlying postharvest physiology and metabolism of fruit and vegetables through multi-omics technologies. Sci. Hortic..

[26] Agut B., Gamir J., Jacas J.A., Hurtado M., Flors V. (2014). Different metabolic and genetic responses in citrus may explain relative susceptibility to Tetranychus urticae. Pest Manag. Sci..

[27] Vogt T. (2010). Phenylpropanoid biosynthesis. Mol. Plant..

[28] Zhang Y., Zhang W., Wang H., Shu C., Chen L., Cao J., Jiang W. (2023). The combination treatment of chlorogenic acid and sodium alginate coating could accelerate the wound healing of pear fruit by promoting the metabolic pathway of phenylpropane. Food Chem..

[29] Wang P., Gong Q., Hu J., Li X., Zhang X. (2021). Reactive Oxygen Species (ROS)-Responsive Prodrugs, Probes, and Theranostic Prodrugs: Applications in the ROS-Related Diseases. J. Med. Chem..

[30] Xue Z., Wang B., Qu C., Tao M., Wang Z., Zhang G., Zhao M., Zhao S. (2023). Response of salt stress resistance in highland barley (*Hordeum vulgare* L. var. nudum) through phenylpropane metabolic pathway. PLoS ONE.

[31] Yan Z., Wang H., Kou X., Wu C., Fan G., Li T., Zhou D. (2022). Metabolomics analysis reveals that MeJA treatment induces postharvest blueberry resistance to Botrytis cinerea. Postharvest Biol. Technol..

[32] Wang M., Li C., Liu J., Zhang S., Guo Y., Jin Y., Ge Y. (2023). Phenylalanine maintains the postharvest quality of ‘Jinfeng’ pear fruit by modulating the tricarboxylic acid cycle and chlorophyll catabolism. Postharvest Biol. Technol..

[33] Aghdam M.S., Jannatizadeh A., Luo Z., Paliyath G. (2018). Ensuring sufficient intracellular ATP supplying and friendly extracellular ATP signaling attenuates stresses, delays senescence and maintains quality in horticultural crops during postharvest life. Trends Food Sci. Technol..

