# Supplementary material for: Untargeted Metabolomics Reveals the Effect of Carbon Dots on Improving the Shelf Life of Postharvest Goji Berries (Lycium barbarum L.)
Source: Foods. 2025 Sep 26;14(19):3336. doi: 10.3390/foods14193336 (PMC12523848; doi:10.3390/foods14193336)
Supplement: Supplementary file 1 [file foods-14-03336-s001.zip › Table S1; Table S2; Table S3..pdf]

Supplemental Table S1. Liquid phase gradient setting parameters

Time (min)	Flow (mL/min)	B%
0.0-0.5	0.3	5
0.5-7.0	0.3	5-100
7.0-8.0	0.3	100
8.0-8.1	0.3	100-5
8.1-11.0	0.3	5

Supplemental Table S2. Mass spectrum parameters

Argument	Set value
Shielding pressure of ion source	0 PSI
Gas 1 (auxiliary gas) pressure	10 PSI
Gas 2 (sheath gas) pressure	35 PSI
Source temperature	350°C
Positive ion mode time voltage	4000 v
Negative ion mode time voltage	-4500

Supplemental Table S3. Differential metabolites and identification results

Scan patterns	all	Identification of metabolites		
		Secondary metabolites	Primary metabolites	
			KEGG	HMDB
pos	12231	220	5656	6355
neg	5369	230	2449	2690
